# Retreatment with Immune Checkpoint Inhibitors in the New Scenario of Immunotherapy in Non-Small Cell Lung Cancer

**DOI:** 10.3390/cancers16091683

**Published:** 2024-04-26

**Authors:** Sabrina Rossi, Silvia Masini, Giovanna Finocchiaro, Elena Lorenzi, Luca Toschi, Armando Santoro

**Affiliations:** 1 Medical Oncology and Hematology Unit, IRCCS Humanitas Research Hospital, Via Alessandro Manzoni 56, Rozzano, 20089 Milan, Italy; sabrina.rossi@humanitas.it (S.R.); giovanna.finocchiaro@humanitas.it (G.F.); elena.lorenzi@humanitas.it (E.L.); luca.toschi@humanitas.it (L.T.); armando.santoro@humanitas.it (A.S.); 2 Department of Biomedical Sciences, Humanitas University, Via Rita Levi Montalcini 4, Pieve Emanuele, 20072 Milan, Italy

**Keywords:** non-small cell lung cancer, immunotherapy, rechallenge

## Abstract

**Simple Summary:**

First-line treatment for metastatic, non-oncogene-addicted non-small cell lung cancer primarily relies on immune checkpoint inhibitors. Recently, immunotherapy has significantly improved clinical outcomes in patients with early-stage and locally advanced disease, leading to its approval in these settings. However, the management of patients who have relapsed after treatment with immune checkpoint inhibitors with curative intent appears to be more challenging. This review examines the current state of knowledge about rechallenge with immunotherapy.

**Abstract:**

The advent of immunotherapy has transformed the treatment paradigm for metastatic non-small cell lung cancer (NSCLC). In the past few years, several studies have investigated the potential role of immune checkpoint inhibitors (ICIs) in resectable and unresectable locally advanced disease, achieving remarkable results that led to their approval in clinical practice. However, there is limited evidence on immunotherapy rechallenge after recurrence, with the majority of available knowledge coming from retrospective studies which involve heavily pretreated patients with advanced NSCLC. The recent introduction in the curative setting and the potential regulatory restrictions raise questions about the optimal choice of first-line and subsequent therapies for patients with systemic relapse. The role of immunotherapy readministration in this new scenario needs to be clarified, as well as the identification of patients for whom it is more appropriate, including clinical characteristics, duration of response, switching to other ICIs, reasons for discontinuation and immune-related toxicity. Here, we review literature on rechallenge with immunotherapy, including efficacy, safety profile and potential predictive factors of response.

## 1. Introduction

Lung cancer stands as the foremost cause of cancer-related deaths globally [1]. Recent years have witnessed a revolutionary shift in the treatment of metastatic non-small cell lung cancer (NSCLC) with the advent of immune checkpoint inhibitors (ICIs). After the results observed in several clinical trials [2,3,4,5], ICIs, including anti-programmed death receptor-1 (PD-1), anti-programmed death ligand-1 (PD-L1) and anti-cytotoxic T-lymphocyte-associated protein-4 (CTLA-4) antibodies, have become a standard of care in first-line treatment of non-oncogene-addicted metastatic NSCLC as a monotherapy or in combination with platinum-based chemotherapy, according to PD-L1 status [6,7,8,9,10,11].

Immunotherapy is also acquiring an increasingly important role in the treatment of early-stage and locally advanced NSCLC. The phase III PACIFIC trial was the first pivotal study investigating the role of durvalumab, an anti-PD-L1 antibody, as consolidation treatment in locally advanced unresectable stage III NSCLC after concomitant chemoradiation therapy (CRT) [12]. A significant improvement in progression-free survival (PFS) was observed in the durvalumab arm compared to the placebo arm, confirmed at 5-years follow-up, with a sustained overall survival (OS) of 47.5 months in the experimental arm vs. 29.1 months with placebo [13]. A post-hoc subgroup analysis showed that PD-L1 expression was measurable in 63% of patients, and durvalumab did not improve OS outcomes in patients with a negative PD-L1 expression, although PFS was still remarkable in this subgroup [14]. Therefore, durvalumab was approved by the European Medicines Agency (EMA) for patients with unresectable stage III PD-L1 positive NSCLC without disease progression (PD) after concurrent/sequential CRT. The recent results from the PACIFIC-6 trial have confirmed the efficacy of durvalumab after sequential chemoradiation therapy in PD-L1-positive patients [15].

Recently, ICIs have also been approved as adjuvant treatment after chemotherapy, in completely resected stage II-IIIA. In the Impower010 trial, atezolizumab was evaluated after adjuvant platinum-based chemotherapy in patients with completely resected stage IB–IIIA NSCLC [16], showing a disease-free survival (DFS) improvement compared to the best supportive care in PD-L1 ≥ 1% tumors; notably, in PD-L1 ≥ 50% population, the reduction of risk in disease progression was 57%. The pre-specified exploratory OS analyses were conducted in the intention-to-treat (ITT) population (stage IB-IIIA), in stage II-IIIA and in patients with stage II-IIIA NSCLC with PD-L1 ≥ 1%: a greater benefit was observed in patients with stage II-IIIA disease and PD-L1 ≥ 1%. In addition, post-hoc analyses were conducted in the PD-L1 ≥ 50%, 1–49% and <1% subgroups in stage II-IIIA population, showing major benefits in patients with PD-L1 ≥ 50%, leading to the approval of adjuvant atezolizumab only in this subpopulation.

Pembrolizumab demonstrated significant DFS benefits in patients with IB-IIIA resected NSCLC, regardless of PD-L1 levels [17]. Even though chemotherapy was not mandatory in the pivotal trial, pembrolizumab is indicated as an adjuvant treatment following platinum-based chemotherapy.

In the last few years, ICIs have also been investigated in a neoadjuvant setting. CheckMate 816 was the first open-label phase 3 trial in which patients with resectable stage IB to IIIA NSCLC were randomized to receive neoadjuvant nivolumab plus chemotherapy, or chemotherapy alone followed by surgery [18]. Patients with epidermal growth factor receptor (EGFR) or anaplastic lymphoma kinase (ALK) alterations were excluded; PD-L1 status was assessed in the overall population. Nivolumab plus chemotherapy showed a significantly longer event-free survival (EFS) compared to the chemotherapy alone (*p* = 0.005) and a higher percentage of patients with a pathological complete response (pCR; *p* < 0.001). Given the results observed in the CheckMate 816 trial, nivolumab gained EMA approval as a neoadjuvant treatment in combination with chemotherapy in patients with resectable NSCLC and PD-L1 expression ≥ 1%. Other recent pivotal studies demonstrated the efficacy of perioperative immunotherapy. KEYNOTE-671 is a randomized, double-blind, phase 3 trial evaluating perioperative pembrolizumab in patients with early-stage NSCLC: 786 patients with resectable stage II, IIIA or IIIB (N2 stage) NSCLC were randomized to receive neoadjuvant pembrolizumab or placebo with cisplatin-based chemotherapy for four cycles, followed by surgery and adjuvant pembrolizumab or placebo for one year [19]. This study met both primary endpoints of EFS and OS [20]. The CheckMate 77T trial evaluated neoadjuvant nivolumab plus chemotherapy followed by adjuvant nivolumab with a significant improvement in pCR, major pathological response (MPR) and EFS compared to chemotherapy plus adjuvant placebo (*p* = 0.00025) [21]. In the AEGEAN trial, 802 patients with stage II-IIIB NSCLC were randomized to receive durvalumab or placebo plus platinum-based chemotherapy for four cycles before surgery, then adjuvant durvalumab or placebo for 1 year; the pCR rate was significantly higher for the experimental arm (*p* = 0.00003) and EFS was significantly prolonged in the durvalumab plus chemotherapy arm (*p* = 0.003) [22].

Despite the improvements in early-stage treatments, locoregional and distant recurrences are frequently observed [13,23]. With the increasing use of ICIs in earlier stages of NSCLC, the therapeutic algorithm of relapsed metastatic disease should be redefined, as well as the role of ICIs in this setting. Data about the effectiveness of ICI rechallenge in patients already treated with immunotherapy in early-stage disease are still limited. The aim of this review is to examine the evidence available on the rechallenge of ICIs in the setting of early stage, locally advanced and metastatic disease.

## 2. Immunotherapy Rechallenge in Advanced/Metastatic Disease

Some data about the rechallenge of ICIs in the metastatic setting are available from prospective studies, as immunotherapy was administered for up to 2 years in most pivotal clinical trials. In KEYNOTE-024, the authors reported outcomes in patients who received a second course of pembrolizumab after completing 35 cycles as first-line treatment and experiencing disease progression: four out of 12 patients had an objective response after starting a second course of pembrolizumab, all partial responses (PRs), and six had stable disease (SD) as their best response [24]. In the KEYNOTE-010 study, the analysis of long-term outcomes also included 14 patients who completed 35 cycles/2 years of pembrolizumab and received a second course of pembrolizumab (up to 17 cycles): six patients had a PR, and five had an SD as best response [25].

Additional data came from the 5-year analysis of KEYNOTE-042, in which 33 patients received a retreatment with pembrolizumab after completion of 35 cycles: five patients experienced a PR and 20 an SD, with a disease control rate (DCR) of 75.8% [26].

A phase I/II trial conducted in patients with various solid tumors investigated the efficacy of retreatment with durvalumab in patients who had disease control and stopped durvalumab after one year of treatment as per protocol [27]. Out of 71 patients who received a retreatment, 21 were affected by NSCLC. In the overall population, 11.4% of patients achieved a PR, and 60% an SD. The median duration of response (DOR) was 16.5 months, and the most prolonged response was 25.1 months in one patient affected by NSCLC. The DCR during the retreatment period at 24 months was 47.1% in the whole population and 33.3% in NSCLC patients.

A pooled analysis assessed the outcomes of a second course of pembrolizumab in NSCLC patients treated with pembrolizumab alone (cohort 1) or combined with chemotherapy (cohort 2) as a first-line treatment in phase 3 trials [28]. This analysis included patients who progressed after completing 35 cycles of pembrolizumab with at least SD as best response or stopped pembrolizumab before the completion of 2 years of treatment due to CR. At rechallenge, the median PFS was 8.2 months in cohort 1 and 7.7 months in cohort 2, with a 6-months PFS rate of 59.6% and 58.3%, respectively. The median OS was 27.5 in cohort 1, while, in cohort 2, it was not reached at the time of analysis; the OS rate at 6 months was 85.4% and 86.2% in the two cohorts, respectively. The overall response rate (ORR) during retreatment was 19% in cohort 1 and 6% in cohort 2: an SD was observed in 31 (53.4%) patients in cohort 1 and in seven (43.8%) in cohort 2. In addition, retreatment with pembrolizumab showed a DCR of 72.4% and 50% of patients in cohorts 1 and 2, respectively. Such encouraging evidence was found in a population that received immunotherapy rechallenge after completing a defined course of treatment and had received fewer lines of cancer-related therapies.

A single-arm, phase 3 trial evaluated the safety and efficacy of atezolizumab in patients with NSCLC who had received up to two lines of systemic treatment, including chemotherapy alone or in combination with anti-PD-1 antibodies, and anti-PD-1 monotherapy [29]. The study enrolled 101 patients; 97 of them were included in the evaluable population. Previous therapy with anti-PD-1 agents was administered to eight patients. In the overall population, the median OS was 15.3 months (95% CI, 11.33–18.60 months), and the median PFS was 2.8 months (95% CI, 2.66–4.21 months). The ORR was 16.5% (with one patient achieving CR and 15 patients PR) and the median DOR was 16.8 months. As expected, the OS benefit was lower in patients who had previously received an anti-PD-1 inhibitor as monotherapy (4.5 months) or combined with chemotherapy (9.6 months) than in those who had not previously received immunotherapies (15.6 months).

Most data on immunotherapy rechallenge in metastatic setting come from retrospective studies conducted with heavily pretreated patients receiving ICI monotherapy in late lines of treatment.

Sun et al., conducted a retrospective analysis to assess outcomes in patients who stopped immunotherapy after 2 years and those who continued beyond 2 years of treatment [30]. The study enrolled 1091 patients, with 113 patients in the fixed-duration cohort and 593 in the indefinite-duration cohort. In the fixed-course group, 10 (8.8%) patients had a negative PD-L1 expression, 25 (22.2%) a PD-L1 expression of 1–49%, and 51 (45.1%) ≥ 50%. The first treatment was immunotherapy in 59 (52.2%) patients and chemoimmunotherapy in 54 (47.8%) patients. Rechallenge with ICIs was delivered in 11 patients after PD in the fixed-duration cohort: eight received an ICI as monotherapy and three were treated with an ICI in combination with chemotherapy. Among them, 10 were retreated with the same ICI. After ICI rechallenge, the median PFS was 8.1 months.

In a retrospective cohort, 40 patients were rechallenged with ICIs as monotherapy or combined with other agents [31]. As prior treatment, 53% of patients received an anti-PD-1 agent plus chemotherapy (mainly a platinum-based regimen), 25% of patients received an anti-PD-1 as monotherapy, 13% of patients received an anti-PD-1 combined with an angiogenesis inhibitor, and 10% received a triplet of an anti-PD-1 plus a chemotherapy regimen plus an angiogenesis inhibitor. After progression to the first immunotherapy, 83% of patients were directly retreated with ICIs as subsequent therapy. Most patients were rechallenged with combination regimens, 43% with an anti-PD-1/anti-PD-L1 plus chemotherapy, 25% with an anti-PD-1 plus angiogenesis inhibitors, and 25% with an anti-PD-1 in combination with chemotherapy and angiogenesis inhibitors. Only three (8%) patients were retreated with immunotherapy alone, and 17 (43%) patients received a different ICI. The median PFS was 6.8 months (95% CI 5.8–7.8 months). OS data were immature. PR and SD were observed in nine (22.5%) and 25 (62.5%) patients, respectively. The ORR was 22.5%, and the DCR was 85%.

A report of 12 patients previously treated with nivolumab investigated the efficacy of retreatment with pembrolizumab [32]. All patients discontinued nivolumab due to disease progression; the median PFS on nivolumab was 6.2 months. Cytotoxic chemotherapy was administered between nivolumab and pembrolizumab in eight (66.7%) cases. The median PFS with pembrolizumab was 3.1 months: four patients had SD as their best response and only one patient achieved PR.

Another retrospective study examined retreatment with ICIs in 11 patients [33]. All patients had received nivolumab first, then a rechallenge with nivolumab was administered in 10 patients, only one patient was treated with pembrolizumab. In most cases (10 out of 11), chemotherapy was administered between the first course of immunotherapy and rechallenge; the DCR was 45%, and the median PFS was 2.7 months.

In a small series of 13 patients with various cancers who had discontinued ICIs in phase I trials as per protocol with a tumor-controlled disease (including CR, PR or SD), the authors reported the results of retreatment with the same immunotherapy at the time of progression [34]. After discontinuation of the first ICI, disease progression occurred in eight patients in a median time of 11.7 months. Among them, one patient had NSCLC and achieved a remarkable PFS of 35.4 months during rechallenge with ICIs.

Takahama et al., reported efficacy data on ICI readministration in 10 patients with NSCLC [35]. After progression on a first course of immunotherapy, five patients received pembrolizumab, four received nivolumab, and one received atezolizumab. The response was poor, with three patients having SD and seven experiencing PD as the best response during retreatment. Similar results were observed in a small cohort of 14 patients who were retreated with immunotherapy after PD [36]. In this study, 11 patients received nivolumab, two atezolizumab, and one pembrolizumab as prior treatment. Nivolumab was readministered in nine patients and pembrolizumab in five patients, with eight patients receiving the same type of ICI during rechallenge. The median PFS and OS were 1.6 and 6.5 months, respectively, and the ORR was 7.1%.

Although these results are not entirely consistent, the benefit of a rechallenge with immunotherapy appears limited in patients pretreated with anti-PD-1/anti-PD-L1 agents, probably due to the high number of previous treatments or to the development of resistance mechanisms to ICIs. Data from these studies are summarized in Table 1.

## 3. Immunotherapy Rechallenge after ICI Treatment in Early-Stage or Locally Advanced Disease

The updated 5-years analysis from PACIFIC trial reported data about anticancer therapies administered after discontinuation of durvalumab and time to first (TFST) and second (TSST) subsequent therapy or death. In the experimental arm, 48% of patients received at least one subsequent anticancer therapy, most commonly chemotherapy (33.0%), followed by radiotherapy (20.4%) [13]. Subsequent immunotherapy was less commonly administered in patients treated with durvalumab (12.6%): 37 patients received nivolumab and 16 patients received pembrolizumab. Authors reported improvement in both TFST and TSST with durvalumab compared to the placebo, but no further data are available about the outcomes of patients who received ICIs as a subsequent line of therapy. Other systemic therapies were administered in 11.1% of patients, including tyrosine kinase inhibitors (TKIs).

In the IMpower010 study, the authors reported on post-recurrence systemic therapies [23]. In the atezolizumab arm, 24.1% of patients required at least one subsequent systemic treatment and 5.7% of patients received immunotherapy, including pembrolizumab (19 patients), nivolumab (six patients), durvalumab (two patients), ipilimumab (two patients) and atezolizumab (one patient). Two other patients were treated with a bispecific antibody targeting PD-1 and lymphocyte-activation gene (LAG-3) tebotelimab and THOR-707 (SAR444245), a pegylated recombinant non-alpha IL-2, respectively, administered within clinical trials. However, data on the efficacy and safety of readministration of immunotherapy were not presented. After adjuvant atezolizumab, 18.1% of patients were treated with chemotherapy, 7.5% with TKIs, and 3.9% with monoclonal antibodies. Locoregional treatments were also reported: 5% of patients underwent surgery and 11% received radiotherapy for recurrence in the experimental arm.

Bruni et al., conducted a retrospective study on 238 patients treated with durvalumab after concomitant or sequential CRT for unresectable stage III NSCLC [37]. Median follow-up was 14 months, relapses occurred in 55 out of 238 (23%) patients, and 30 out of 55 (54.5%) patients received a subsequent treatment (26 with chemotherapy and four with pembrolizumab). Locoregional recurrences occurred in 5.8% of patients, while 29.7% of patients developed distant metastases. Patients with a local recurrence were treated with stereotactic radiotherapy, while most of patients with distant metastases received a systemic therapy or best supportive care. Survival data did not include subgroup analysis for patients treated with immunotherapy.

A real-world prospective analysis evaluated the efficacy and treatment patterns after durvalumab maintenance in 26 patients who received concurrent or sequential CRT for unresectable and locally advanced NSCLC and PD-L1 expression ≥ 1% [38]. Patients with locoregional relapse or oligoprogression received a local treatment (radiotherapy or surgery), while patients with a systemic recurrence were treated with chemotherapy or targeted therapies (one patient received a RET inhibitor).

Rosner et al., reported the 5-year results of a phase I/II trial investigating the role of nivolumab as a neoadjuvant treatment in 21 patients with early-stage NSCLC [39]. A tumor recurrence was observed in seven patients (four out of seven with stage IIIA NSCLC; four out of seven with a PD-L1 < 1%); intrathoracic recurrences were observed in three (43%) patients. Regarding subsequent therapies, three patients received a locoregional treatment, one patient underwent chemoradiation therapy and two patients were treated with a systemic therapy (one with platinum-based chemotherapy and one ROS1-positive patient with a target therapy). No one received ICIs as a subsequent treatment. Table 2 reports data on retreatment with ICIs in resectable and unresectable locally advanced disease.

## 4. Efficacy Outcomes of Immunotherapy Rechallenge by Switching ICIs

Clinical data about effectiveness of switching administration of anti-PD-1 and anti-PD-L1 antibodies at rechallenge are limited. Fujita et al., conducted a retrospective analysis on 15 NSCLC patients [40]. Among them, 14 received atezolizumab as an initial treatment and one patient received durvalumab as consolidation immunotherapy, after chemoradiation treatment. At disease progression, seven patients received nivolumab and eight pembrolizumab. No patients achieved a PR or a CR, while an SD as best response was observed in three patients treated with pembrolizumab and in one patient receiving nivolumab. The median PFS was 2.8 months with pembrolizumab and 1.9 months with nivolumab.

Similarly, studies evaluating NSCLC patients who had been treated with anti-PD-1 antibodies showed poor efficacy from a subsequent line of treatment with anti-PD-L1 agents. A retrospective study examined outcomes of ICI retreatment in 18 patients who had already been treated with anti-PD-1 inhibitors, including eight patients treated with nivolumab, seven with pembrolizumab and three patients with both nivolumab and pembrolizumab [41]. All patients were rechallenged with atezolizumab. The observed benefit was limited, since seven patients showed an SD, and 11 showed a PD as the best response.

Furuya et al., investigated the efficacy of atezolizumab in 152 patients with advanced, pretreated NSCLC [42]. A total of 38 (25%) patients had already received an anti-PD-1 agent, including nivolumab or pembrolizumab. In these patients, the median time to treatment failure (TTF) of atezolizumab was 2 months, and the ORR and DCR were 2.6% and 34.2%, respectively.

Another report of 35 NSCLC patients who had received and discontinued an ICI treatment due to disease progression confirmed poor response to different immunotherapy agents [43]. In this study, 19 patients received nivolumab, 12 received pembrolizumab and four received atezolizumab as initial immunotherapy. Rechallenge treatment consisted of nivolumab in five patients, pembrolizumab in seven patients and atezolizumab in 23 patients. All patients received a different agent (anti-PD-1 or anti-PD-L1), based on the first line of treatment received. During the retreatment, no patient achieved a CR, only one patient had a PR, 14 patients experienced an SD, and 18 patients experienced a PD (two patients were not evaluable). The ORR was 2.9% and the DCR was 42.9%. Different results were described in a case series in which 10 out of 17 patients achieved a PR or an SD after switching administration of anti-PD-1 to anti-PD-L1 antibodies [44]. Most patients (88.2%) received anti-PD-1 antibodies as prior treatment, including nivolumab and pembrolizumab, and four patients received both anti-PD-1 agents as first and third ICI. Initial treatment with ICIs was discontinued in 10 patients due to PD and in seven patients due to immune-related adverse events (irAEs). At rechallenge, median PFS was 4.0 months, and the median OS was 31.0 months, with two patients still on treatment at the end of the observation period.

In a retrospective analysis, 24 patients with advanced NSCLC were rechallenged with a different ICI [45]. Patients were divided into a responder group (CR, PR or SD as best response) and a non-responder group (PD as best response). There were no significant differences in patient characteristics between the two groups; however, all patients in the responder group had an Eastern Cooperative Oncology Group Performance Status (ECOG PS) of 0–1, none of them had liver metastases or was on steroid therapy, while, in the non-responder group, there were three patients with a ECOG PS of 2 and one patient was receiving steroids for palliative purposes. The switch from initial to a different ICI due to disease progression or irAEs occurred with higher frequency among the responder group compared to the non-responder group (*p* = 0.006). In the responder group, there were two patients with PR and nine with SD. The ORR was 8.3%, and the DCR was 45.8%. However, there was no significant difference in OS between the two groups (*p* = 0.059), but there was a trend towards a longer OS in the responder group.

Finally, a recent meta-analysis investigated the efficacy and safety of immunotherapy rechallenge in NSCLC patients, including the above-mentioned studies [46]. Regarding the strategy of switching agents, Cai et al., found that the readministration of the same agent showed no difference in clinical outcomes compared to the prior treatment (*p* > 0.05), while switching ICIs was associated with a lower efficacy. Such evidence could justify the decision to readminister the same ICI agent; however, this analysis referred to heavily pretreated patients, who are difficult to compare with patients who received immunotherapy in the curative setting. Table 3 resumes the data on switching ICIs at rechallenge.

## 5. Efficacy Outcomes of Immunotherapy Rechallenge Based on Reasons for Discontinuation of Prior ICI Treatment

As already mentioned, reasons for immunotherapy discontinuation include disease progression, occurrence of irAEs and completion of a fixed course of treatment.

The meta-analysis by Cai et al., compared the outcomes of patients receiving immunotherapy rechallenge based on the reason of discontinuation. It showed superior benefit in patients who discontinued prior ICI due to irAEs or clinical decisions [46]. As a matter of fact, the ORR and DCR of ICI rechallenge in patients who had interrupted previous ICI treatment due to progressive disease were 8% and 39%, respectively; on the other hand, one-third of patients who discontinued initial treatment with ICIs due to toxicity or completed a fixed course had an objective response when retreated with ICIs. In addition, a high rate of DCR was observed in this population, reaching 71% of cases. In this context, patients who relapse after completing a consolidation treatment with durvalumab or a neoadjuvant/adjuvant immunotherapy may benefit from immunotherapy rechallenge, probably also depending on DFS duration.

## 6. Safety of the ICI Rechallenge

Frequency and severity of irAEs could represent a concern in ICI rechallenge, especially if the cause of the interruption of the first course was immune-related toxicity. Safety data on immunotherapy retreatment are listed in Table 4.

The meta-analysis by Cai et al., reported incidence rates of all-grade and high-grade (defined as grade ≥ 3) irAEs of 41% and 13%, respectively, during retreatment [46]. No difference in incidence rates was found between initial immunotherapy and rechallenge (all-grade: odds ratio 1.42; 95% CI: 0.48–4.19; *p* = 0.53; high-grade: odds ratio 0.80; 95% CI: 0.24–2.69; *p* = 0.72).

At rechallenge, patients may experience the same irAEs developed during the prior immunotherapy or a new irAE. A retrospective analysis reported clinical outcomes and safety in 84 patients who developed irAEs during anti-PD-1 treatment with nivolumab or pembrolizumab [47]. Immunotherapy was continued in 32 patients and stopped in 52 patients. Among them, 14 patients resumed the same anti-PD-1 inhibitor. Recurrent irAEs were observed in two (14%) patients, while two other patients (14%) developed new irAEs. Recurrent grade 3 immune-related toxicity (thyroid dysfunction) was reported in one patient, and ICI was permanently discontinued. There was no significant difference in the incidence of grade ≥ 3 irAEs between patients who resumed immunotherapy and those that discontinued treatment.

Gobbini et al., conducted a retrospective analysis of 144 patients who received immunotherapy rechallenge after ICI discontinuation due to disease progression (40%), immune-related toxicity (40%) and clinical decision (20%) [48]. Among patients who stopped immunotherapy due to toxicity, four patients experienced a grade ≥ 3 irAE during rechallenge, with the same irAE recurring in two cases (pneumopathy) and a completely new irAE in other two cases (arthralgia and nephritis). Anti-PD-1 antibodies were mostly administered during the first course of immunotherapy (88% of patients) and at rechallenge (94% of patients).

A retrospective single-center study analyzed the safety and efficacy of ICI readministration after the onset of irAEs in NSCLC patients treated with anti-PD-1, anti-PD-L1 or anti-CTLA-4 antibodies in combination or as a monotherapy [49]. In the analysis, 99 patients were included, and, in 40 cases, immunotherapy was readministered after a first course. Patients who received another anticancer therapy between discontinuation of immunotherapy and retreatment were excluded. The median time between the onset of irAEs and the resumption of ICI therapy was 59 days. Immune-related toxicities were observed in 24 patients, with 16 (66.7%) patients having the same irAE and eight (33.3%) having a de novo irAE. Grade 3 irAEs occurred in seven (29.2%) patients, while no patients experienced grade 4 irAEs. The most common adverse events of any grade were colitis, rash, hepatitis and pneumonitis.

Another study reported the safety of immunotherapy rechallenge in 17 patients with metastatic NSCLC treated with a first course of anti-PD-L1 or anti-PD-1 [44]. The reason for treatment discontinuation was disease progression in 10 (58.9%) patients and toxicity in seven (41.1%) patients. Among them, two patients were still receiving the second course of ICI treatment at the end of the observation period. Grade 3 pneumonitis recurred during the second ICI treatment and improved with glucocorticoids. However, one patient died from newly diagnosed pneumonitis at rechallenge.

Mouri et al., investigated the feasibility of the readministration of nivolumab in NSCLC patients who had stopped an anti-PD-1 therapy due to the occurrence of irAEs [50]. In this study, 49 patients were enrolled and 21 received a retreatment with nivolumab, while 28 permanently discontinued treatment. The first course of immunotherapy was discontinued due to grade 3–4 adrenal insufficiency in two patients, pneumonitis in one patient, liver dysfunction in one patient, colitis in one patient, and rash in one patient. All severe immune-related toxicities resolved to grade 0–1 irAEs, with 15 (71.4%) patients requiring steroid therapy. During the rechallenge period, only one patient experienced a grade ≥ 3 irAE (colitis) and five patients were treated with steroids.

Similarly, another retrospective study investigated the safety and efficacy of immunotherapy rechallenge in NSCLC patients after the onset of irAEs: in this analysis, 24 out of 38 patients (63%) patients received anti-PD-1 or anti-PD-L1 as monotherapy, while 14 (37%) patients received a combination with an anti-CTLA-4 as initial immunotherapy [51]. During the first treatment, among 38 patients who were retreated with ICIs, 13 (34%) patients experienced a grade 3–4 immune-related toxicity. The median time from first irAE to retreatment was 32 days. No subsequent irAEs were observed in 18 patients, the same irAE recurred in 10 (26%) patients, and 10 (26%) patients developed a completely new irAE. Grade 3–4 irAEs occurred in eight out 20 (40%) patients, with six out of eight experiencing a relapse of the same grade 3–4 irAE and two patients developing a new grade 3–4 irAE. Most irAEs (85%) that occurred during retreatment were manageable and resolved or improved to grade 1. However, two treatment-related deaths occurred; in particular, one patient who developed grade 3 lipase elevation as a first irAE died of colitis, and one patient who discontinued treatment with anti-PD-1 due to grade 2 colitis died of pneumonitis.

Encouraging results on the safety of a retreatment were also observed in patients with various solid tumors retreated with durvalumab [27]. Grade 3–4 irAEs occurred in 5.7% of patients, consisting of transaminase elevation, hyperglycemia, pancreatitis and pneumonitis (1.4% each). Severe irAEs were observed in two (2.9%) patients (pancreatitis and pneumonitis, respectively). Treatment-related deaths were rare during a second course of immunotherapy [27,42,44,45,49].

A meta-analysis conducted by Xu et al., including most of the above-mentioned studies showed that the incidence of grade 3–4 irAEs was lower with retreatment compared to initial immunotherapy (8.6% vs. 17.8%, *p* < 0.001) [52].

Given these findings, the treatment with a second course of immunotherapy is feasible from a safety perspective, based on the grading and the pattern of irAEs.

## 7. Possible Predictors of Response to Immunotherapy Rechallenge

Patient characteristics may affect response to immunotherapy rechallenge.

Furuya et al., observed that patients with a high expression of PD-L1 (≥50%) had a longer median OS and TTF than patients with lower or negative PD-L1 expression, but no statistically significant differences were evidenced [42].

A good performance status and a longer duration of the first ICI treatment have also been shown to positively impact OS [42,48,53]. In contrast with this evidence, Xu et al., found no statistical correlation between PFS and performance status, retreatment with the same ICI or not and PD-L1 expression [31].

A multicenter, retrospective study analyzed the correlation between baseline patient characteristics and OS in 18,186 patients treated with ICIs alone or combined with chemotherapy or target therapy, including 12,416 patients with NSLCL [54]. OS was significantly longer in patients with high levels of basal albuminemia and elevated eosinophil and lymphocyte counts (*p* < 0.0001).

Other factors that had a positive predictive effect on PFS under rechallenge (PFSR), but were not statistically confirmed, were the reason for the first discontinuation of ICI (longer PFSR was observed in irAEs or clinical decision when compared to progressive disease), no systemic treatment between the two courses of ICIs and rechallenge in early-line settings [48]. The onset of irAEs could be related to a better response to immunotherapy. A pooled analysis investigated the association between irAEs and immunotherapy efficacy in NSCLC patients [55]. The study included Impower130, Impower132 and Impower150, three randomized phase III trials enrolling 2503 chemotherapy naïve patients that evaluate the safety and efficacy of atezolizumab combined with chemotherapy and/or bevacizumab. In the atezolizumab-containing arm, the median OS was 25.7 months in patients with irAEs vs. 13.0 months in those without irAEs (HR 0.69; 95% CI 0.60–0.78), and the ORR was 61% vs. 37%, respectively. Patients with mild irAEs (grade 1–2) experienced a greater benefit in terms of survival compared to both patients who reported no irAEs or severe irAEs. Even if these findings relate to patients treated with first-line ICIs, the occurrence of mild irAEs may be a factor to consider when evaluating whether a patient could benefit from a rechallenge.

Additional data come from a multicenter retrospective study of 134 patients with metastatic NSCLC treated with second-line or later nivolumab [56]. IrAEs occurred in 69 (51%) patients, and a significant improvement in clinical outcomes was observed compared to patients without irAEs, with a PFS of 9.2 vs. 4.8 months (*p* = 0.04) and OS of NR vs. 11.1 months (*p* = 0.01), respectively. However, the authors did not specify if patients had already received immunotherapy.

In patients who have completed a fixed course of immunotherapy, time to progression might be an aspect to consider. In the KEYNOTE-010 study with a longer follow-up, 23 out of 79 patients completed 35 cycles or 2 years of pembrolizumab and experienced PD during the follow-up period; 14 patients were retreated with pembrolizumab [25]. Median PFS was not reached (95% CI, 14.3 months to NR) and PFS rates at 12 and 24 months after treatment completion were 72.5% (95% CI, 59.9% to 81.8%) and 57.7% (95% CI, 41.2% to 71.0%), respectively.

In the new scenario where early immunotherapy is expected, the treatment-free interval could be considered when selecting patients who might benefit from rechallenge. Schoenfeld et al., recommended the readministration of ICI in patients who had progressed after at least 6 months since the last treatment [57]. A phase II trial evaluating the efficacy of rechallenge with nivolumab in 61 patients with metastatic NSCLC who had responded to previous immunotherapy and had received the last dose of ICI at least 60 days before demonstrated that an ICI-free interval > 9.2 months significantly correlated with a longer PFS [58].

## 8. Discussion

The therapeutic scenario of NSCLC is rapidly evolving thanks to the efficacy demonstrated by immunotherapy. The remarkable results achieved with ICIs have led to the approval of these agents in curative setting, both for unresectable stage III NSCLC, for resectable locally advanced or early-stage disease. Given the favorable data from the PACIFIC and Impower010 studies, several trials have been conducted to investigate the role of immunotherapy in unresectable stage III disease and as adjuvant treatment [17,59,60,61]. The efficacy of immunotherapy was also demonstrated as a neoadjuvant and perioperative treatment in combination with chemotherapy in several pivotal trials [18,19,21,22].

The promising improvements in DFS and EFS offer hope for the increased potential of curing patients with immunotherapy in the early-stage setting. A meta-analysis by Guven et al., comparing neoadjuvant chemoimmunotherapy to standard neoadjuvant chemotherapy, demonstrated a 41% reduction in the risk of progression or death (HR: 0.59, 95% CI: 0.52–0.66, *p* < 0.0001) [62]. However, the current data have limited follow-up, making estimating the proportion of patients cured with resectable disease challenging. On the other hand, 33.1% of patients with unresectable stage III lung cancer who have received consolidation with durvalumab are progression-free at 5 years [13].

The use of ICIs in non-metastatic NSCLC raises questions about the choice of subsequent therapies for tumor progression. Disease relapse was the most common cause of death in the patients enrolled in the PACIFIC trial, both in the durvalumab group (43.7%) and in the placebo group (49.8%), as well as in the IMpower010 study, with 63% of patients in the atezolizumab arm and 80% in the control arm dying from disease relapse [13,23]. Therefore, proper treatment planning is crucial and the pattern of relapse should be considered when choosing a new line of therapy.

Patients with local or oligometastatic recurrence may receive local treatment such as radiotherapy or surgery, as suggested during multidisciplinary tumor board discussions [13,16,37,38,39]. On the other hand, when driver mutations are detected in metastatic settings, the standard first-line treatment is represented by targeted therapies [63,64]. However, in the future, it is possible that a complete molecular profile of NSCLC patients will be available in earlier stages due to the raising interest in the use of targeted therapies as neoadjuvant or adjuvant treatment and the scarce benefit of immunotherapy in oncogene-addicted patients.

Currently, the first-line therapy for non-oncogene-addicted metastatic NSCLC is immunotherapy alone or in combination with chemotherapy, based on PD-L1 expression [64,65]. Patients eligible for consolidation with durvalumab or perioperative immunotherapy have already been selected for positive PD-L1 expression. However, PD-L1 is a biomarker characterized by spatial and temporal heterogeneity. Its expression can differ within the same tumor specimen as well as between primary tumor and metastases [66]. PD-L1 expression is also dynamic over time, depending on disease course and anticancer treatments received [67]. In none of the studies investigating the efficacy of rechallenge with immunotherapy was the PD-L1 status re-evaluated. In patients who have relapsed after completing a course of immunotherapy, the option of a new tissue biopsy or a liquid biopsy for molecular analysis could be considered. It would be interesting to explore whether PD-L1 changes over time correlate with a benefit from ICI retreatment.

The feasibility of ICI readministration may be limited due to regulatory indications and reimbursement requirements, as they are high-cost therapies and data on rechallenge in this new scenario are limited. The modalities of approval and access to anticancer treatment, the timing of reimbursement and the assessment of the economic impact of drugs on the healthcare budget differ not only between the FDA and the EMA but also between different European countries [68]. The urgency of determining the potential benefit of retreatment and access to immunotherapy for selected patients cannot be overstated, as immunotherapy for localized disease will be the standard of care in the near future.

Data available on retreatment with immunotherapy are limited and come mainly from retrospective studies conducted in heavily pretreated patients with metastatic disease. According to the meta-analysis by Cai et al., ICI rechallenge showed a lower efficacy compared to the responses obtained during the first course of immunotherapy [46]. However, endpoints of these studies were ORR and DCR, which are not applicable to disease-free NSCLC patients. The efficacy of readministration was higher in patients who discontinued the prior treatment due to irAEs or after completion of a fixed course than in patients who interrupted a previous immunotherapy due to progressive disease [24,25,27,28,46]. Patients who received immunotherapy in a non-metastatic setting could be similar to those who discontinue their first course of treatment after a fixed number of cycles, for whom rechallenge data are more encouraging.

However, the reported studies included patients with different demographic and clinical characteristics, including age, gender, race, smoking history, histology and PD-L1 expression, and it is not possible to directly compare the results, which is a relevant limitation in interpreting the available data.

Several ongoing trials are investigating the safety and efficacy of anti-PD-(L)1 in combination with innovative drugs that have a potential synergistic effect with ICIs, including immune-modulatory small molecules and monoclonal antibodies, an anticancer vaccine, a fecal microbiota transplant and gene therapy, in patients already treated with immunotherapy (NCT03977467, NCT04691817, NCT05467748, NCT03600701, NCT04919369, NCT05599789, NCT04263051, NCT05669846, NCT04911166 and NCT05334329).

New immune checkpoints are emerging in immunotherapy for solid tumors, including coinhibitory and costimulatory molecules. The first category includes the lymphocyte activation gene 3 (LAG-3), T cell immunoglobulin and mucin domain-3 (TIM-3) and T cell Ig and ITIM domain (TIGIT), which are associated with resistance to conventional immunotherapy and have attracted considerable interest, leading to their evaluation in various solid tumors alone or in combination with anti-PD-(L)1 [69,70,71,72]. The inducible T cell costimulator (ICOS), OX40 and 4-1BB are costimulatory molecules that enhance the T cell-mediated immune response and represent promising targets [73,74]. Several ongoing trials are evaluating immunotherapy rechallenge after prior ICIs alone or in combination with novel compounds targeting emerging immune checkpoints in the metastatic setting (NCT05325684, NCT04655976, NCT04725188, NCT03977467, NCT03697304).

Looking ahead, these new molecules could potentially play a role as subsequent lines of treatment in patients with NSCLC who relapse after receiving ICIs for early-stage disease.

The most effective agent that could be used as rechallenge therapy is still not clear, whether some data support the use of the same ICI at recurrence [46]. ICI readministration seems manageable both in patients who had progressed on previous immunotherapy and in those who had discontinued it due to the onset of irAEs [40,44,48,50,51]. Although current guidelines recommend permanent discontinuation for severe irAEs [75,76], in the literature, some cases of patients with grade 3–4 irAEs retreated with an ICI are described, with most immune-related toxicities regressing to grade 0–1 after steroid therapy. Therefore, in patients who have experienced irAEs during the first course of immunotherapy, retreatment could be considered in select cases, carefully weighing the risks and benefits.

Identifying factors that may lead to the appropriate selection of patients who could benefit from rechallenge is crucial. However, subgroup analyses examining the correlation between patient characteristics and outcomes, including PD-L1 expression, smoking status and histological subtype, were inconclusive [31,42,48,53].

Patients treated with immunotherapy for localized disease with an ICI-free interval and who experienced irAEs may be good candidates for retreatment. However, the optimal duration of treatment-free survival is unclear, and the evidence comes mainly from patients who have previously received immunotherapy for advanced disease.

Future research should focus on trials which explore the correct algorithm of treatment after metastatic progression, investigating the use of the same ICI in advanced settings or the switching strategy, and the possible predictive factors for response to rechallenge also for metastatic patients. There is a need for a more comprehensive knowledge of the mechanisms underlying disease progression in patients who discontinued ICIs for reasons other than disease progression, such as completing the treatment course or experiencing unacceptable toxicity.

## 9. Conclusions

The new algorithm for the treatment of NSCLC, which includes the use of immunotherapy in the early stages, leaves some concerns about the choice of first-line treatment for systemic recurrences. ICI rechallenge could be a feasible and safe strategy for patients who relapse after the first immunotherapy treatment received in the curative setting, but no scientific data definitely validate this hypothesis. Predictive factors of ICI activity in the context of rechallenge should be explored to make an appropriate selection of patients for ICI readministration.

## Figures and Tables

**Table 1:** Immunotherapy rechallenge in advanced/metastatic disease.

Study	Prospective/ Retrospective	ICI First Course	ICI Monotherapy Rechallenge (%)	ICI + CT Rechallenge (%)	PFS (m)	ORR/DCR (%)
KEYNOTE-024 2021 [24]	Prospective	Pembrolizumab	7.8	0.0	-	33.3/83.3
KEYNOTE-010 2020 [25]	Prospective	Pembrolizumab	2.0	0.0	-	42.9/78.6
KEYNOTE-042 2023 [26]	Prospective	Pembrolizumab	5.2	0.0	-	15.2/75.8
Sheth et al., 2020 [27]	Prospective	Durvalumab	42.3	0.0	-	11.4/61.4
Xu et al., 2023 [29]	Prospective	Anti-PD-1	100.0	0.0	1.4 ^a^/2.0 ^b^	-
Sun et al., 2023 [30]	Retrospective	Anti-PD(L)1	7.1	2.6	8.1	-
Xu et al., 2022 [31]	Retrospective	Anti-PD-1	8.0	68.0	6.8	22.5/85.0
Fujita et al., 2018 [32]	Retrospective	Nivolumab	100.0	0.0	3.1	8.3/41.7
Niki et al., 2018 [33]	Retrospective	Nivolumab	100.0	0.0	2.7	27.0/45.0
Bernard-Tessier et al., 2018 [34]	Observational	Anti-PD(L)1	100.0	0.0	12.9	25.0/100.0
Takahama et al., 2018 [35]	Retrospective	Anti-PD(L)1	4.7	0.0	-	0.0/30.0
Watanabe et al., 2019 [36]	Retrospective	Nivolumab Atezolizumab Pembrolizumab	4.4	0.0	1.6	7.1/21.4

^a^ Median PFS in patients treated with a first course of ICI as monotherapy. ^b^ Median PFS in patients treated with a first course of ICI combined with CT. ICI: immune checkpoint inhibitor; CT: chemotherapy; PFS: progression-free survival; ORR: overall response rate; DCR: disease control rate; Anti-PD(L)1: anti-programmed death receptor (or ligand)-1.

**Table 2:** Immunotherapy rechallenge after ICI treatment in early-stage or locally advanced disease.

Study	Retrospective/ Prospective	ICI First Course	ICI at Relapse (%)	CT at Relapse (%)	TKIs (%)	Locoregional Treatments at Relapse (%)
PACIFIC 2022 [13]	Prospective	Durvalumab	Nivolumab (7.8) Durvalumab (7.1) Pembrolizumab (3.3)	33.0	11.1 ^a^	20.4
IMpower010 2023 [23]	Prospective	Atezolizumab	Pembrolizumab (2.0) Nivolumab (<1) Atezolizumab (<1) Durvalumab (<1)	14.0	6.0	16.0
Bruni et al., 2021 [37]	Retrospective	Durvalumab	Pembrolizumab (7.3)	47.3	0.0	16.3
Taugner et al., 2021 [38]	Prospective	Durvalumab	0.0	7.7	7.7	15.4
Rosner et al., 2023 [39]	Prospective	Nivolumab	0.0	9.5	4.8	19.0

^a^ Including TKIs, among other treatments. ICI: immune checkpoint inhibitor; CT: chemotherapy; TKIs: tyrosine kinase inhibitors.

**Table 3:** Efficacy outcomes of immunotherapy rechallenge by switching ICIs.

Study	Prospective/ Retrospective	ICI First Course	ICI Second Course	PFS (m)	OS ^a^ (m)	ORR/DCR (%)
Fujita et al., 2020 [40]	Retrospective	Atezolizumab Durvalumab	Nivolumab Pembrolizumab	1.9 2.8	-	0.0/14.3 ^b^ 0.0/37.5 ^c^
Fujita et al., 2019 [41]	Retrospective	Nivolumab Pembrolizumab	Atezolizumab	2.9	-	0.0/38.9
Furuya et al., 2021 [42]	Retrospective	Nivolumab Pembrolizumab	Atezolizumab	-	-	2.6/34.2
Katayama et al., 2019 [43]	Retrospective	Nivolumab Pembrolizumab Atezolizumab	Nivolumab Pembrolizumab Atezolizumab	2.7	26.9	2.9/42.9
Kitagawa et al., 2020 [44]	Retrospective	Nivolumab Pembrolizumab Atezolizumab	Nivolumab Atezolizumab	4.0	31.0	5.9/58.8
Takahara et al., 2022 [45]	Retrospective	Anti-PD(L)1	Anti-PD(L)1	-	NE ^d^/30.6 ^e^	8.3/45.8

^a^ From the first ICI treatment. ^b^ DCR with nivolumab. ^c^ DCR with pembrolizumab. ^d^ OS of patients in the responder group. ^e^ OS of patients in the non-responder group. ICI: immune checkpoint inhibitor; PFS: progression-free survival; OS: overall survival; ORR: overall response rate; DCR: disease control rate; Anti-PD(L)1: anti-programmed death receptor (or ligand)-1; NE: not evaluated.

**Table 4:** Safety of the ICI rechallenge.

Study	ICI First Course	Grade ≥ 3 irAEs at First Course (%)	ICI Rechallenge	Same irAEs (%) All Grades	Different irAEs (%) All Grades	Grade ≥ 3 irAEs at Rechallenge (%)	Type of Grade ≥ 3 irAEs at Rechallenge
Fujisaki et al., 2021 [47]	Nivolumab Pembrolizumab	11.2	Nivolumab Pembrolizumab	14.3	14.3	7.1	Thyroid dysfunction
Gobbini et al., 2020 [48]	Anti-PD(L)1	18.7	Anti-PD(L)1	-	-	6.2	Pneumopathy Arthralgia Nephritis Colitis Not specified
Guo et al., 2022 [49]	Nivolumab Pembrolizumab Nivolumab + ipilimumab	49.5	Nivolumab Pembrolizumab Nivolumab + ipilimumab Atezolizumab	40.0	20.0	17.5	Pneumonitis Colitis Dermatitis Hepatitis
Kitagawa et al., 2020 [44]	Nivolumab Pembrolizumab Atezolizumab	17.6	Nivolumab Atezolizumab	23.5	17.6	11.8	Pneumonitis
Mouri et al., 2019 [50]	Nivolumab	33.3	Nivolumab	61.9	19.0	4.7	Colitis
Santini et al., 2018 [51]	Anti-PD(L)1 +/− anti-CTLA-4	34.0	Anti-PD(L)1 +/− anti-CTLA-4	26.0	26.0	21.0	Colitis Hepatic failure Pneumonitis Not specified
Takahara et al., 2022 [45]	Anti-PD(L)1	16.7	Anti-PD(L)1	4.7	8.3	4.7	Pneumonitis

ICI: immune checkpoint inhibitor; irAEs: immune-related adverse events; Anti-PD(L)1: anti-programmed death receptor (or ligand)-1; anti-CTLA-4: anti-cytotoxic T-lymphocyte-associated protein-4; ALT: alanine aminotransferase.
